# Angiomatoid Fibrous Histiocytoma, A Great Mimicker –A Short Series of 3 Cases with EWSR1 Fusion

**DOI:** 10.30699/ijp.2023.558035.2937

**Published:** 2023-02-03

**Authors:** C Aparna Devi, Ashwini Nargund, Geeta V Patil Okaly, Usha Amirtham

**Affiliations:** Department of Pathology, Kidwai Memorial Institute of Oncology, Rajiv Gandhi University of Health Sciences, Bengaluru, Karnataka, India

**Keywords:** Angiomatoid fibrous histiocytoma, EWSR1 fusion, Fibrous histiocytoma

## Abstract

Angiomatoid Fibrous histiocytoma (AFH) is a rare soft tissue neoplasm that is often misdiagnosed initially. It is commonly encountered in the superficial extremities of children and young adults. It is composed of a nodular proliferation of bland looking spindled to ovoid cells, some with variant histology and characterized by EWSR1 fusion. We, herein, present three such cases, who presented with swelling in the right leg (case 1), right forearm (case 2), and right thigh (case 3). Case 2 presented in the fourth decade with a large swelling compared to the other two cases that presented in 3rd decade with a small swelling. Histologic examination of case 2 showed extensive myxoid changes making it diagnostically challenging. All three cases showed EWSR1 fusion with a break-apart probe. Follow-up was uneventful in all three cases. AFH, although it is a benign neoplasm, is a great mimicker of various low-grade spindle cell sarcomas. Awareness of this entity with its various histomorphological variants is necessary to accurately diagnose this lesion.

## Introduction

In the past decade, angiomatoid fibrous histiocytoma (AFH) was considered one of the five subtypes of malignant fibrous histiocytoma (now classified as undifferentiated pleomorphic sarcoma) (1). It is an uncommon fibroblastic/myofibroblastic neoplasm of intermediate biologic potential with rarely metastasizing characteristics. It is commonly encountered in the superficial extremities of children and young adults (2). AFH is usually located in the deep dermis and subcutis and carries a good prognosis (3). It was initially described by Enzinger and his colleagues in 1979, wherein they described it through a series of 41 cases (4). Through this short series of three cases of young Indian adults who presented to our institute – Kidwai Memorial Institute of Oncology, Bengaluru, Karnataka, we intend to discuss the diagnostic challenges of this rare entity encountered histologically and immunohistochemically.

## Case Presentation

**Case 1**

**Fig. 1:**
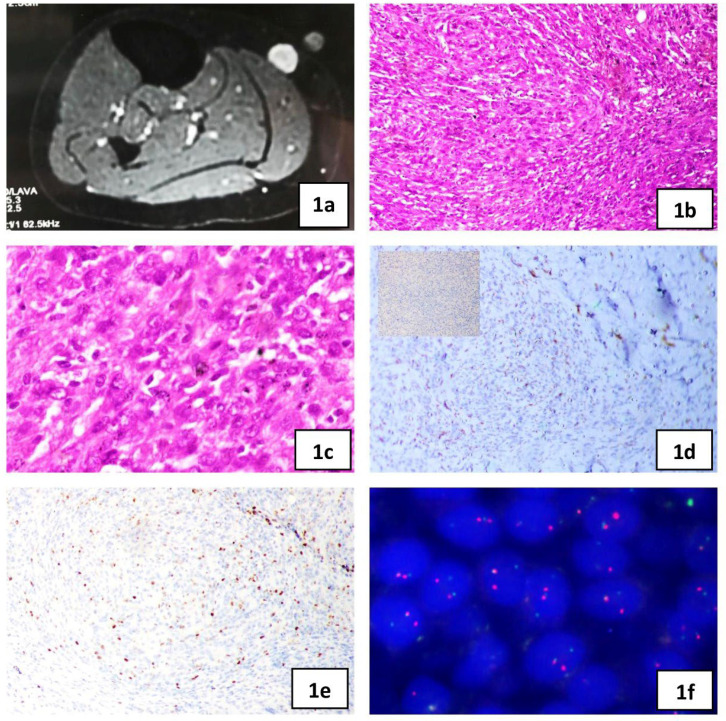
**Case 1) **a. MRI leg showing hyperintense lesion on T2WI, b. The vague nodular proliferation of spindle cells, H&E x100, c. Plump spindle cells with a vesicular nucleus, H&E ×400, d. CD68, ×100, Inset: Desmin, x100, e. Ki67, ×100, f. FISH for EWSR1 showing break-apart red and green signals

 A previously healthy 21-year-old female with no comorbid illness or significant family history presented in October 2020 with complaints of swelling in the right leg for 6 months. It was insidious in onset, without much progression in size, but was associated with pain. The patient was moderately built and nourished with stable vitals and systemic functions. On local examination, a small 0.5- 1cm tender nodule was palpable on the anteromedial aspect of the upper third of the right leg. The skin over the nodule was smooth and normal. Initial blood investigations were within normal limits. Magnetic Resonance Imaging (MRI) of the right leg showed a well-defined lesion measuring 12×10×8 mm in the subcutaneous plane of the anteromedial aspect of the mid-leg, which is hypointense on T1 and hyperintense on T2 weighted images (T2WI). The lesion showed subtle central T2 hypo intensity and avid enhancement on contrast. It was seen to abut the medial head of gastrocnemius with loss of fat planes. There were no abnormal intermuscular signal changes/vascular compression/ marrow infiltration. MRI features were suggestive of a peripheral nerve sheath tumor (Figure 1a).

With prior informed consent, Fine needle aspiration cytology (FNAC) of the swelling was performed, and showed atypical, spindled cells and was reported positive for malignancy. Further histological evaluation was advised. The patient underwent wide local excision of the swelling. The serial slicing of the skin-covered soft tissue mass revealed a solitary well-circumscribed grey–white nodular lesion measuring 1×1×1.2 cm with a homogenous cut surface without any foci of necrosis or hemorrhage. The overlying skin was unremarkable. Multiple sections examined from the lesion showed a well-circumscribed encapsulated neoplasm composed of oval to spindle-shaped cells arranged in fascicles, sheets, and storiform pattern with peripheral lymphoid cuffing (Figure 1b). The cells exhibited moderate nuclear pleomorphism with vesicular nuclear chromatin (Figure 1c). No areas of necrosis or hemorrhage were noted. Mitotic activity was counted as 8/10 hpf. All margins were free of tumors. On immunohistochemistry, scattered neoplastic cells were immunoreactive for CD68 (Figure 1d) and TLE1. They were negative for desmin (Figure1d, inset), S100, Smooth Muscle Actin (SMA), Pancytokeratin (PanCK), CD45, ERG, CD99, CD34 and ALK. The ki67 index was 10% (Figure 1e). Fluorescence in situ Hybridisation (FISH) was positive for EWSR1 rearrangement by using a DNA break-apart probe (CytoTest Inc.) at chromosome band 22q12.2 (Figure 1f). The patient was followed up for 19 months till March 2022 and was fine.

**Case 2**

A 36-year-old male came to us in February 2017 with complaints of a non-tender swelling in the lateral aspect of his right forearm of 17 months duration, which was insidious in onset and gradually progressed to a bigger size. He was a well-built and nourished person with no significant comorbidities or family history and had a stable general examination. Upon examination of the swelling, the swelling measured approximately 5×3cm with a bosselated surface. The skin over the swelling was pinchable. Motor and sensory functions of the right upper limb were normal and distal pulsations were felt. All other systems and initial blood investigations were within normal range. MRI of the right forearm showed a well-defined lobulated soft tissue intensity lesion measuring 5 x 4 x 3.3 cm in the anterolateral aspect of the flexor compartment along the tendon of flexor carpi radialis distally and reached up to the subcutaneous plane anterolaterally. It was hypointense on T1 and hyperintense on T2WI. A few small cystic areas were noted within. There was no evidence of bone erosion/ destruction. The lesion was seen to abut the radial artery with loss of fat planes. MRI features were suggestive of soft tissue neoplasm with the possibility of synovial sarcoma. Doppler's study of the right upper limb did not show involvement of the radial artery. The lesion was seen close to the nerve and was thought to be arising from the nerve. After obtaining informed consent from the patient, further investigations were performed.

**Fig. 2:**
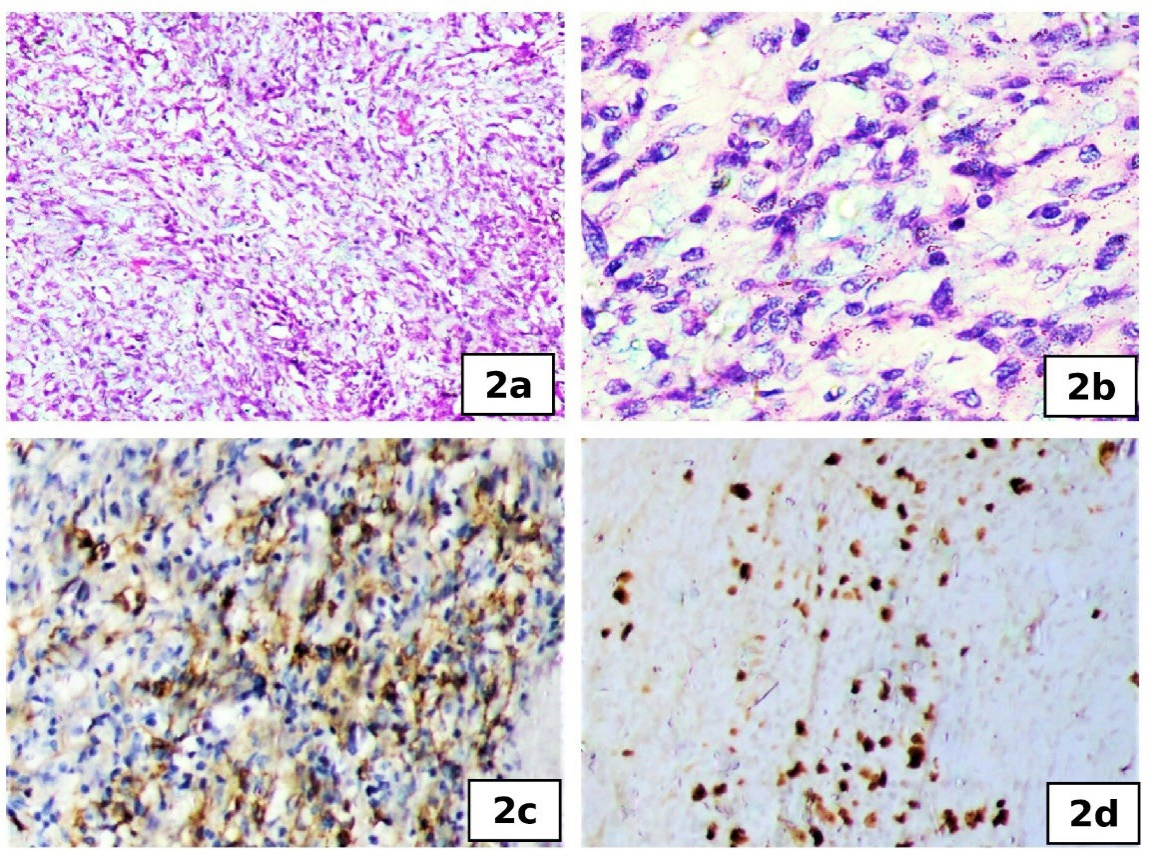
**Case 2) **a. H&E ×100, b. spindled to ovoid cells, H&E ×400, c. CD68, ×100, d. Ki67, ×100

FNAC of the lesion showed oval to spindle cells in clusters and sheets with bland nuclear features and focal myxoid areas. A Trucut biopsy of the swelling showed cores of the tumor with varying cellularity composed of normochromatic spindle cells with indistinct margins and scant cytoplasm. Occasional atypical cells were noted (Figure 2a, b). Myxoid changes were noted. Necrosis and mitotic activity were not observed. The neoplastic cells were immunopositive for Epithelial Membrane Antigen (EMA), CD99, BCL2, TLE1, and PanCK and were not reactive for CD34, SMA, S100, and desmin. Correlating with radiology, a possibility of synovial sarcoma was considered and advised excision. The wide local excision specimen consisted of a skin-covered soft tissue mass. The overlying skin was unremarkable. On serial slicing, a circumscribed encapsulated lesion was seen in the subcutaneous plane measuring 6.5x5x2.5cm. The lesion was lobulated with a grey - white soft cut surface. Multiple sections from the lesion revealed a spindle cell neoplasm with areas of myxoid changes and hyalinization. Many congested vessels and aggregates of lymphoid cells were noted in the periphery of the lesion. There was no evident necrosis, and mitotic activity was counted as 10/10 hpf. The overlying skin and all resected margins were free of tumors. Patchy immunoreactivity of neoplastic cells was noted for CD68 (Figure 2c), EMA, desmin, CD99, and BCL2. They were negative for CK7, S100, MyoD1, CD21 and CD23. Ki67 proliferative index was 10-12% (Figure 2d). A diagnosis of Angiomatoid fibrous histiocytoma with extensive myxoid changes was rendered. The neoplasm was positive for EWSR1 rearrangement at chromosome 22q12.2 by break apart (CytoTest Inc. probe) FISH. The patient did not have any complaints of recurrence or distant lesions during the follow-up period of 61 months.

A summary of case details is provided in Table 1. Immunohistochemical characteristics of the cases are provided in Table 2.

**Case 3**

A well-nourished and moderately built 20-year-old young man presented to our hospital in June 2018 with complaints of swelling associated with pain in the right thigh for 3 months; he had no significant comorbid illness and family history. The swelling did not progress in size significantly. General and systemic examination was within normal limits. Local examination of the swelling revealed it to be a non-tender swelling of size 2 x 2 cm with well-defined margins, and the skin over the swelling was pinchable. Routine blood investigations were within normal hematological and biochemical limits. MRI of the right thigh showed evidence of a well-defined lesion of size 1 x1.2 x 1.4 cm in the anterolateral aspect of the distal end of the right thigh. It was hypointense on T1 and hyperintense on T2WI. The lesion was seen arising from the fascial sheath of the vastus lateralis muscle in the anterolateral aspect of the right thigh. All other structures were within normal radiological limits. MRI findings were highly suggestive of a soft tissue neoplasm and advised histopathological evaluation.

**Fig. 3:**
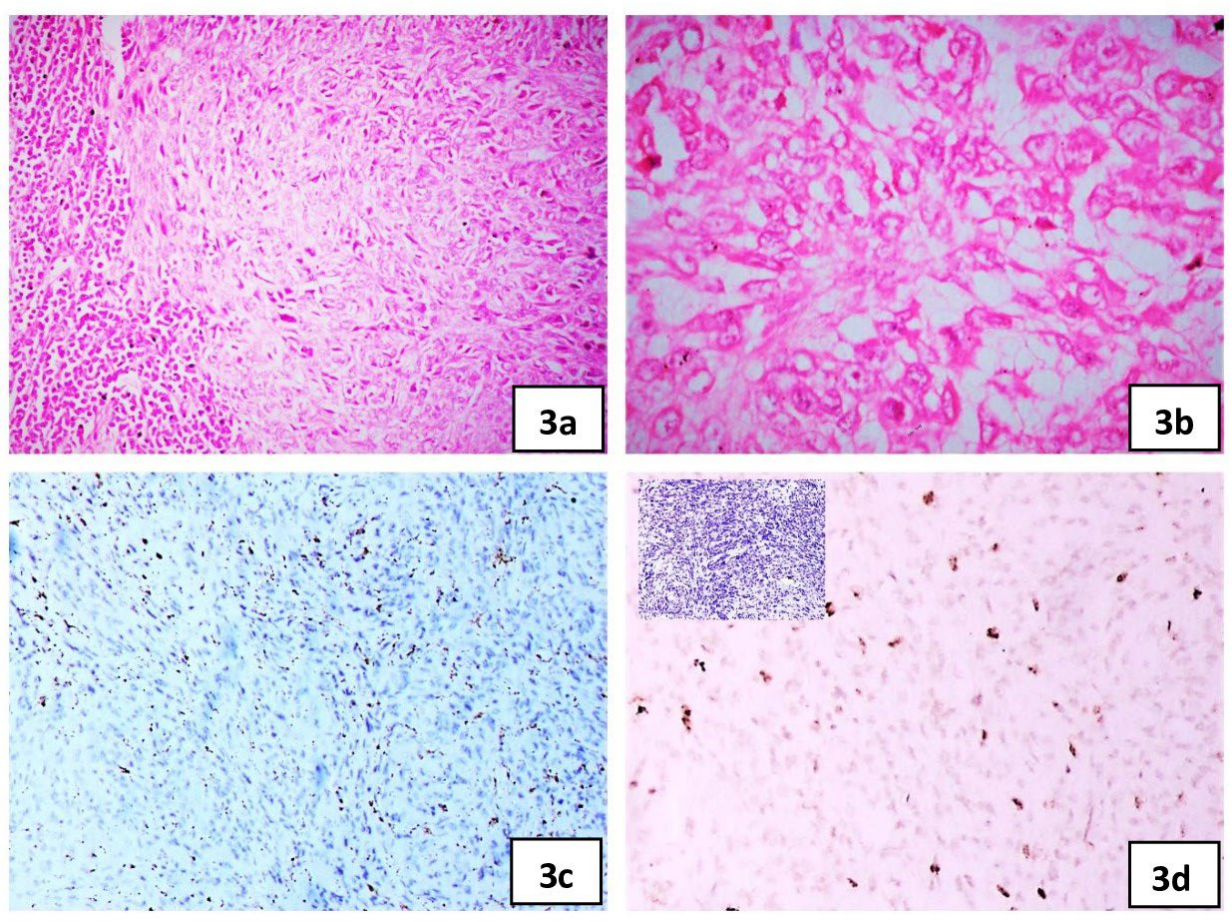
**Case 3) **a. shows spindle cell proliferation with a peripheral rim of lymphoid cells, H&E ×100, b. plump spindled to ovoid cells, H&E ×400, c. CD68, ×100, d. Ki67, ×100, Inset: Desmin, ×100

After obtaining informed consent, wide local excision of the lesion was done. Grossly, it was a well-encapsulated nodular lesion in the subcutaneous plane measuring 2.2 × 2.5 × 2 cm. The cut surface of the lesion was grey–white, homogenous, and firm. Areas of necrosis or hemorrhage were not encountered. Microscopically, the lesion showed a vague nodular and whorled pattern of spindled cells with indistinct cell margins and vesicular nuclei. There was a dense chronic inflammatory cell infiltrate comprising lymphocytes and histiocytes at the periphery of the tumor (Figure 3a, b). Mitotic activity was counted as 7/10 hpf. There was no evidence of cellular pleomorphism, necrosis, or atypical mitosis. On IHC, the neoplastic cells were focally positive for CD68 (Figure 3c), EMA, and SMA. They were negative for PanCK, desmin (Figure 3d inset), CD45, CD34, S100, H – caldesmon, HMB45, synaptophysin, CD21, CD23, MPO, CD1a, BCL2, CD30 and ALK. The ki67 index was 12% (Figure 3d). FISH for EWSR1 gene rearrangement by using a break-apart probe (CytoTest Inc.) on chromosome 22q12.2 was positive. The patient had an uneventful course during the follow-up period of 45 months. 

**Table 1:** Summary of Case Details

Case	Age/Sex	Site	Size	MRI	Cytology	Outcome
Case 1	21 years/ female	Right upper leg (anteromedial)	1×1×1.2 cm	T2 hyperintense lesion abutting the medial head of the gastrocnemius	Atypical spindled cells, positive for malignancy	Follow-up – 19 months, uneventful
Case 2	36 years/male	Right distal forearm (anterolateral)	6.5×5×2.5 cm	T2 hyperintense lesion along flexor carpi radialis tendon	Spindle cell lesion with focal myxoid areas	Follow-up – 61 months, uneventful
Case 3	20 years/male	Right distal thigh (anterolateral)	2.2×2.5×2 cm	T2 hyperintense lesion along vastus lateralis tendon	Not performed	Follow-up – 45 months, uneventful

**Table 2:** Comparison of IHC of the cases

IHC marker	Case 1	Case 2	Case 3
CD68	Positive	Positive	Positive
Desmin	Negative	Positive	Negative
CD99	Negative	Positive	Not performed
PanCK	Negative	Patchy positive	Negative
EMA	Not performed	Positive	Positive
TLE1	Positive	Positive	Not performed
SMA	Negative	Negative	Positive
S100	Negative	Negative	Negative
CD34	Negative	Negative	Negative
ALK	Negative	Not performed	Negative
BCL2	Not performed	Positive	Not performed
Ki67	10%	10-12%	12%

## Discussion

An angiomatoid fibrous histiocytoma is a locally recurring neoplasm with rarely metastasizing potential. It accounts for only 0.3% of all soft tissue tumors although it may have been misdiagnosed for a variety of other fibroblastic soft tissue lesions (2). Usually, AFH presents as a superficial subcutaneous lesion and is mistaken for a lymph node or a benign cystic lesion clinically (5). It is encountered most commonly in the extremities of people in the first three decades (6).

AFH is a rare neoplasm of intermediate biologic potential presenting in the skin of the upper and lower limbs (7,8). Uncommon sites include breast (9), intrapulmonary origin (10), and intracranial origin (11). Although these represent unusual sites, all of these cases exhibited EWSR1 fusion, which is characteristic of AFH. 

Radiologically, AFH presents as a heterogenous mass with multiple cystic spaces, enhancing fibrous pseudocapsule and foci of blood elements, but most of the time, the findings are non–specific (12). All the cases in our study showed heterogenous hyperintense T2WI. 

In a study of 21 cases by Huijuan Shi *et al*. and another study of 13 cases by Yu – Chien Kao *et al*., all patients presented with painless slow-growing mass, most commonly on the extremities, followed by the trunk without prior history of trauma or constitutional symptoms (13,14). However, in the oldest series of 41 cases by Enzinger, a history of antecedent trauma and constitutional symptoms were elicited in some of the cases (4). Unusually, patients can also present with paraneoplastic syndrome. In a case report by Lerraughn M. Morgan *et al*., the eight-year-old girl presented with chronic bleeding secondary to storage pool platelet dysfunction (15).

A clinicopathological study of 158 cases by Fanburg Smith *et al.* described four main histological characteristics of this lesion, namely the presence of fibrous pseudocapsule, nodular fibrohistiocytic spindled to ovoid cell proliferation, pseudo-angiomatoid spaces, and peripheral lymphoplasmacytic response (3). Many series have also documented the presence of myxoid, edematous changes, and sclerosis (14). Rarely, multinucleated giant cells like Touton giant cells, even focal necrosis, and the absence of classical peripheral lymphoid cuffing have been identified in a few studies (13). Unusual histological features like schwannoma/perineurioma-like areas, small round cells reminiscent of Ewing sarcoma, rhabdomyoblast-like cells, nuclear grooving, and clear cell change have also been documented. Occasional cases have also been shown to have increased mitotic activity (16,17). Case 2 showed extensive myxoid changes adding to the diagnostic confusion, and all 3 cases demonstrated mildly increased mitotic activity in this short series. Case 1 revealed classic histological features. 

Immunohistochemically, the neoplastic spindle cells show variable immunoreactivity for vimentin, CD68, and desmin. Desmin positivity is observed in approximately 50 – 60% of cases indicating myoid differentiation (3). Other markers include CD99 and EMA. Rarely NSE and synaptophysin expression has been demonstrated. Case 1 and case 3 were negative for desmin and case 2 was positive for desmin. Case 2, in addition to extensive myxoid changes, displayed a variable expression for different immunomarkers, including BCL2 and TLE1, leading to the misdiagnosis of synovial sarcoma. So, whenever a relatively well-circumscribed fibrohistiocytic lesion is encountered in the extremities, a diagnosis of AFH should also be considered in the differential, keeping in mind its varied histological patterns and immunoreactivity. 

SOX9, a transcription factor involved in the embryogenesis of cartilage and gonadal tissue, has been expressed in 85% of AFH and is quite specific for it (18).

 Ultrastructurally, AFH cases showed two types of cells. One was histiocytic with irregular nuclei and prominent nucleoli with branching Rough Endoplasmic reticulum (RER) and primary lysosomes. The other was spindle cells with moderate amounts of RER and mitochondria (1). 

A series of five cases from Mumbai with EWSR1 – CREB1 fusion in a single case is one of the few case series from India (19). Molecular studies have revealed specific translocation in AFH involving the EWSR1 family genes. The most common ones are t(12;22), t(2;22) and t(12;16) leading to EWSR1/ATF1, EWSR1-CREB1 and FUS/ATF1 fusion transcripts respectively (20). This family of translocations is also identified in a few other spindle cell malignancies like clear cell sarcoma and myxoid liposarcoma. Although these fusions are diagnostic for AFH, studies evaluating the utility of FISH for ESWR1 and FUS rearrangements demonstrated that a small minority can still be negative for translocations despite cryptic rearrangements (21). Testing for EWSR1 rearrangement is vital as this is a neoplasm with markedly variable histomorphology and immunohistochemical features. 

AFH has a good prognosis, but rare studies have demonstrated local recurrences in a few cases (3,7,4). Nevertheless, the recurrence rate has been reported to be 2% (22,23). Due to this minor chance of local recurrence, all AFH are treated with wide excision. Even cases who have undergone unplanned resections or were misdiagnosed initially should undergo additional extended resections (24). Follow-ups for 19 months, 61 months, and 45 months were available for case 3, case 2, and case 1, respectively, and all were uneventful. 

Even rarer is the occurrence of metastasis in AFH. Although designated as an indolent lesion with variable histology, it is important to remember their metastatic potential (25). 

## Conclusion

AFH is an indolent neoplasm with the potential for local recurrence and metastasis. A correct diagnosis is important for appropriate management. It is misdiagnosed initially due to variable histomorphology like extensive myxoid changes or sclerosis or the absence of classic histological features like a peripheral lymphoid cuff. Immunohistochemistry is also of little help as they may overlap with other spindle cell lesions and show patchy expression. A molecular study for EWSR1 family translocations can aid in the diagnosis. Through this short series, we intend to remind the presence of this rare lesion with overlapping and variable histology and potential for local recurrence.

## Conflict of Interest

Nil.

## Funding

Nil.
